# Iron (III) emissions from seawater desalination plants: a new, hitherto overlooked contribution to the iron cycle in coastal seas?

**DOI:** 10.1007/s10534-026-00861-3

**Published:** 2026-08-07

**Authors:** Frithjof C. Küpper, Mohammed Khalid Massoud, Eleni Avramidi, Ricardo Cruz-López, Kyoko Yarimizu, Martin C. Feiters, Vasilis Louca, Dimitrios Xevgenos

**Affiliations:** 1 School of Biological Sciences, University of Aberdeen, Cruickshank Building, St. Machar Drive, Aberdeen, AB24 3UU Scotland, UK; 2 Oceanography Center, University of Cyprus, 1 Panepistimiou Av., 2109 Aglandjia, Nicosia, Cyprus; 3 Marine Biodiscovery Centre, Department of Chemistry, University of Aberdeen, Aberdeen, AB24 3UE Scotland, UK; 4 Department of Biology, San Diego State University, San Diego, CA 92182-1030 USA; 5 Marine Botany Research Group, Instituto de Investigaciones Oceanológicas (IIO), Universidad Autónoma de Baja California (UABC), 22870 Ensenada, Baja California Mexico; 6 Microbial Genomics and Ecology, PHIS, The IDEC Institute, Hiroshima University, 1-3-2, Kagamiyama, Higashi-Hiroshima City, Hiroshima 739-8511 Japan; 7 Institute for Molecules and Materials, Radboud University, Heyendaalseweg 135, 6525 AJ Nijmegen, The Netherlands; 8 TU Delft, Technology, Policy & Management Faculty, Jaffalaan 5, 2628 BX Delft, The Netherlands

**Keywords:** Brine, Cyprus, Iron(III), Iron limitation, Siderophores

## Abstract

Due to the poor solubility of iron (III) and strong regional differences in terrestrial, riverine or airborne input, iron is a limiting micronutrient in much of the world ocean. Because of increasing living standards and climate change, rainfall is becoming more intermittent, which exacerbates drought conditions. As a result, seawater desalination has a vital and growing role in providing drinking water in many hot and arid regions of the world. Most seawater desalination plants use ferric or ferrate salts as flocculants for removing phyto- and bacterioplankton and suspended organic matter prior to the actual desalination process. Consequently, such seawater desalination plants release large amounts of suspended iron in conjunction with organic matter into coastal waters, with potentially significant consequences for the availability of this micronutrient in coastal marine ecosystems. This review paper provides important background information about the role of iron as a micronutrient in marine ecosystems, its role in the inorganic biochemistry of marine life, as well as key features of its use in seawater desalination, before providing a perspective by exploring potential implications for the physiology and biochemistry of marine life and marine ecosystem functioning.

## Iron in the ocean and as a micronutrient for marine life

Iron, although an important biometal to life, has low bioavailability because of its oxidation, in oxygenated seawater, to poorly soluble iron(III) (Fe(III), Fe^3+^), which at the slightly alkaline pH of seawater precipitates as Fe(OH)_3_ (Liu and Millero 1999; Crichton 2018). The marine biogeochemistry of iron has been extensively reviewed (Shaked et al. 2025 for a recent review). In many parts of the World Ocean, Fe availability is further limited due to missing riverine or wind-borne (aeolian) input (Moore et al. 2006; Raiswell et al. 2008; Shaw et al. 2008; Cropp et al. 2013; Madhusoodhanan et al. 2024). Iron is a micronutrient essential for photosynthesis and nitrogen fixation in marine phytoplankton (Rubin et al. 2011). Following the hypothesis by Martin and Fitzwater (1988) that primary production in large parts of the ocean is limited by a lack of iron—even when macronutrients such as nitrogen and phosphorus are plentiful—so-called HNLC (high nitrogen, low chlorophyll) regions were identified (Minas et al. 1986). To test this hypothesis, large-scale iron fertilization experiments were conducted. These began in the equatorial Pacific (Martin et al. 1994; Coale et al. 1996; Cooper et al. 1996) and later expanded to the Southern Ocean (Boyd et al. 2000; Hoffmann et al. 2006), the Subarctic Pacific (Boyd et al. 1996; Kudo et al. 2005), and the Humboldt Current region off northern Chile (De los Ríos-Escalante et al. 2020). Even small changes in dissolved iron concentrations can strongly influence algal productivity (Van Leeuwe et al. 1997). For example, hydrothermal vents in the deep sea are a major iron source and were recently found to trigger massive phytoplankton blooms in such areas in the Southern Ocean (Ardyna et al. 2019). An interesting aspect of the oceanic cycle of iron and other trace metals is that viruses are potential catalysts of trace metal regeneration through virus-mediated mortality as a source of bioavailable Fe and other metals (Shaked et al. 2025). In coastal and shelf seas, runoff or discharge containing ferric chloride (FeCl_3_) or its hydrolysis products may thus alter nutrient balances, potentially triggering harmful algal blooms (HABs) (Yarimizu et al. 2022). Generally, Fe enrichment can favor certain species that produce toxins harmful to marine life and humans (Sunda 2006; Yarimizu et al. 2022). Furthermore, excess iron concentrations, coupled with coagulant residues or altered pH, can become toxic to algae and other marine organisms (Sunda 2006).

## Anthropogenic iron enrichment and potential impacts

An question occupying scientists and other stakeholders since the late 1980 s is whether large-scale iron fertilization is an option to mitigate anthropogenic CO_2_ emissions and climate change. Despite high hopes, the approach remains unproven and controversial for being a viable and credible strategy at the large scale from the standpoint of biological oceanography and biogeochemistry (Baar et al. 1990; Joos et al. 1991a, 1991b; Peng and Broecker 1991; Zeebe and Archer 2005). One overview (De Baar et al. 2005) highlights the need to be able to extend fertilization experiments for longer time periods (several months), another more recent one (Ward et al. 2025) emphasizes the need for more thorough characterization of oceanographic parameters before techno-economic effects can be assessed.

Accidental, local iron enrichment has been found to have profound effects on certain, normally iron-poor marine ecosystems. For example, the large amount of iron released from ship wrecks in coral reefs of the Line Islands in the HNLC tropical Pacific has been found to result in a phase shift from coral- towards seaweed-dominated communities, characterized by a high abundance of turf algae, macroalgae, cyanobacterial mats and corallimorphs, as well as particulate-laden, cloudy water (Kelly et al. 2012). The routine operations of the global maritime shipping industry provide a pervasive and chronic form of unintended nutrient enrichment including iron. A primary pathway is atmospheric deposition from fuel combustion, which releases highly soluble iron aerosols. One modeling study indicated that this source contributes 30–60% of all soluble iron deposited over the high-latitude, iron-limited North Atlantic and North Pacific (Ito 2013). This effect is often magnified by the co-deposition of nitrogen from the same exhaust plumes (Zhang et al. 2021). Direct aqueous discharges are also a major source. The widespread adoption of exhaust gas cleaning systems (scrubbers) results in a hot, acidic effluent (Shi et al. 2023) that contains significant concentrations of dissolved iron (EPA 2011). Other operational wastewaters are similarly iron-rich: a physico-chemical analysis found that bilge water contained iron concentrations as high as 3.98 mg/L (John et al. 2023), while ballast water acts as a vector for translocating iron-laden coastal sediments (Tolian et al. 2020). Α significant flux stems from the continuous electrochemical and microbially influenced corrosion of steel hulls (Liu et al. 2023). This continuous line source delivery (Pan et al. 2021) is analogous to natural fertilization, which has been shown to be more biologically effective at stimulating marine ecosystems compared to episodic inputs (CBD 2009).

While iron(III) enrichment within the marine environment is widely recognized to promote primary productivity due to the micronutrient nature of iron, aspects of iron enrichment on the physiology of important groups of marine organisms and ecological effects remain understudied. While iron(III) enrichment within the marine environment is widely recognized to promote primary productivity due to the micronutrient nature of iron, aspects of iron enrichment on the physiology of important groups of marine organisms and ecological effects remain. Further to the limited understanding that we have on the impacts on marine algae (Van Leeuwe et al. 1997; Kelly et al. 2012), nothing is known on the cascading effects within marine communities and food webs. Within the plankton communities, there is some limited evidence that the structure of these communities and their species composition is linked to iron availability (Caputi et al. 2019). There is also some evidence that iron (III) availability potentially influences microbial communities, with well recorded increases in primary productivity and consequently heterotrophic bacterial abundance (Cary 2001).

## Inorganic biochemistry of iron in marine organisms 

For a better understanding of potential biological and environmental impacts of iron enrichment, this section provides an introduction to the inorganic biochemistry of iron to marine life. An important aspect of iron which determines its biological role to a large extent is that the iron(II)/iron(III) couple easily undergoes one electron redox reactions at reduction potentials (vs. standard hydrogen electrode at pH 7.0) that can be tuned by the protein environment in the range relevant for biological systems, e.g. − 0.7 to − 0.2 V for ferredoxins that contain iron-sulfur clusters, and − 0.4 to + 0.4 V for cytochromes with iron in the form of heme (Liu et al. 2014). This makes iron-containing proteins indispensable in biological processes involving electron transfer chains, such as oxidative phosphorylation and the aforementioned photosynthesis and nitrogen fixation (Crichton 2018). In these processes, iron cooperates with other metals for certain steps, such as copper for the electron transfer to molecular oxygen in respiration, manganese for the oxygen evolution in photosynthesis, and molybdenum or vanadium in the multi-iron cofactor directly interacting with molecular nitrogen (Crichton 2018). The coordination sphere of the iron may not be completely occupied by protein ligands, so that additional ligands can be bound; these can exchange electrons with the metal, as in the case of transport, as in hemoglobin, or even activation, as in cytochrome-c oxidase, of molecular oxygen, or undergo non-redox rearrangement, as for example in the mitochondrial enzyme cis-aconitase, where the iron just acts as a Lewis acid (Crichton 2018).

Given the natural shortage of iron in the sea, marine life forms have evolved strategies to cope with deficiency, consisting of highly efficient uptake (Sutak et al. 2020; Martín-Barranco et al. 2021; Lampe et al. 2024), recycling (Boyd et al. 2017) and storage systems (Briat et al. 2010; Cruz-López and Carrano 2023), or of replacing iron altogether by using chemical alternatives (Browning et al. 2021; Zhang et al. 2024). Bacteria, fungi (*e.g.* the baker’s yeast *Saccharomyces cerevisiae*), and some plants (such as the monocotyledon grasses, ‘Strategy II’ plants; Müller 2023) acquire iron by excreting small organic ligands called siderophores, and actively taking up the complex with iron in its predominantly occurring state, iron(III), with selective receptors. Siderophores of marine bacteria typically show exceptional affinity for iron (III) compared to their counterparts in the terrestrial and symbiotic (e.g., gut microflora) context (Vraspir and Butler 2009). The strong association with the siderophores provides a driving force to break up the interactions of iron(III) with weaker organic ligands and colloids that it can be associated with. Interestingly, there is not only competition between organisms in the synthesis of chelators with high affinities, but also ‘siderophore piracy’ (Hopkinson and Barbeau 2012) in the sense that organisms can take up complexes of iron(III) with siderophores not synthesized by themselves but by other organisms (Sandy and Butler 2009; Crichton 2018). The existence of systems for the uptake of iron in its most common biological cofactor, heme, has also been demonstrated for marine bacteria (Hopkinson et al. 2008). The competition is such that organisms also express receptors for the complexes with siderophores other than the ones synthesized by themselves (Crichton 2018). Another mechanism of iron uptake, first discovered in the fungus *Saccharomyces* but also present in phytoplankton (Lampe et al. 2024) and ‘Strategy I’ plants (Müller 2023), features reduction of extracellular iron(III) by a ferric reductase at the cell surface, followed by either uptake as Fe(II) through a non-selective divalent metal ion transporter, or more selectively by reoxidation to iron(III) with an oxidase coupled to a ferric iron permease. Interestingly, a number of marine bacterial siderophores containing an α-hydroxycarboxylic acid moiety show photoreactivity, in which the ligand undergoes oxidative decarboxylation with concomitant formation of Fe(II), which is more available to other marine life such as eukaryotic phytoplankton and potentially macroalgae via the aforementioned divalent metal ion transporter. Such photoreactive siderophores include aquachelins (Barbeau et al. 2001, 2003; Barbeau 2006), aerobactin (Küpper et al. 2006), petrobactin (Barbeau et al. 2002) and its sulfonated derivative (Hickford et al. 2004) and vibrioferrin (Amin et al. 2009). The latter is not only photoreactive, but also binds borate, providing an intriguing biochemical link between the biogeochemical iron and boron cycles in the ocean (Amin et al. 2007; Harris et al. 2007; Carrano et al. 2009). Another mechanism of iron uptake by phytoplankton is the synergistic complexation of iron(III) and carbonate by the membrane protein phytotransferrin (Mcquaid et al. 2018), followed by uptake of the complex by endocytosis reminiscent of the uptake by cells of the iron complex of transferrin, the iron transport protein in mammalian plasma (Mcquaid et al. 2018). In iron-limited open ocean waters of the Subantarctic, phytoplankton communities acquire iron bound to strong organic complexes (Maldonado et al. 2005).

While ferritins are the typical iron stores in most pro- and eukaryotes (Ford et al. 1984; Harrison and Lilley 1989; Marchetti et al. 2009; Briat et al. 2010), heterokont organisms are an exception. Centric diatoms (Armbrust et al. 2004) and brown algae, such as the filamentous brown alga *Ectocarpus*, which was the first fully sequenced multicellular alga (Cock et al. 2010, 2012, 2014), do not possess ferritin. In our work, we found an unusual, non-ferritin model of intracellular iron storage in *Ectocarpus* (Böttger et al. 2012) and a large iron(III) store in the brown algal cell wall (Miller et al. 2014). Phaeodactylum and other pennate diatoms are an exception—their ancestors are thought to have acquired ferritin genes by lateral gene transfer (Bowler et al. 2008).

## Seawater desalination

Seawater desalination has an important and growing role in providing drinking, irrigation and process water, especially in arid parts of the world such as the Mediterranean and Middle East, but also in California, Australia, many islands in the world and even countries with seemingly plentiful rainfall such as the Netherlands (Avramidi et al. 2022) and the UK (Rosely and Voulvoulis 2025). Desalinating seawater comes chiefly with two major environmental impacts: Since the process is energy intensive and much of the world’s energy supply remains reliant on fossil fuels despite an urgent need to decarbonize, this comes with an often significant carbon footprint (Cornejo et al. 2014; Xevgenos et al. 2021). The other major impact stems from the discharge of the salt removed from the water, usually in the form of a concentrated brine back into shallow coastal seas (Xevgenos et al. 2021).

**Fig. 1:**
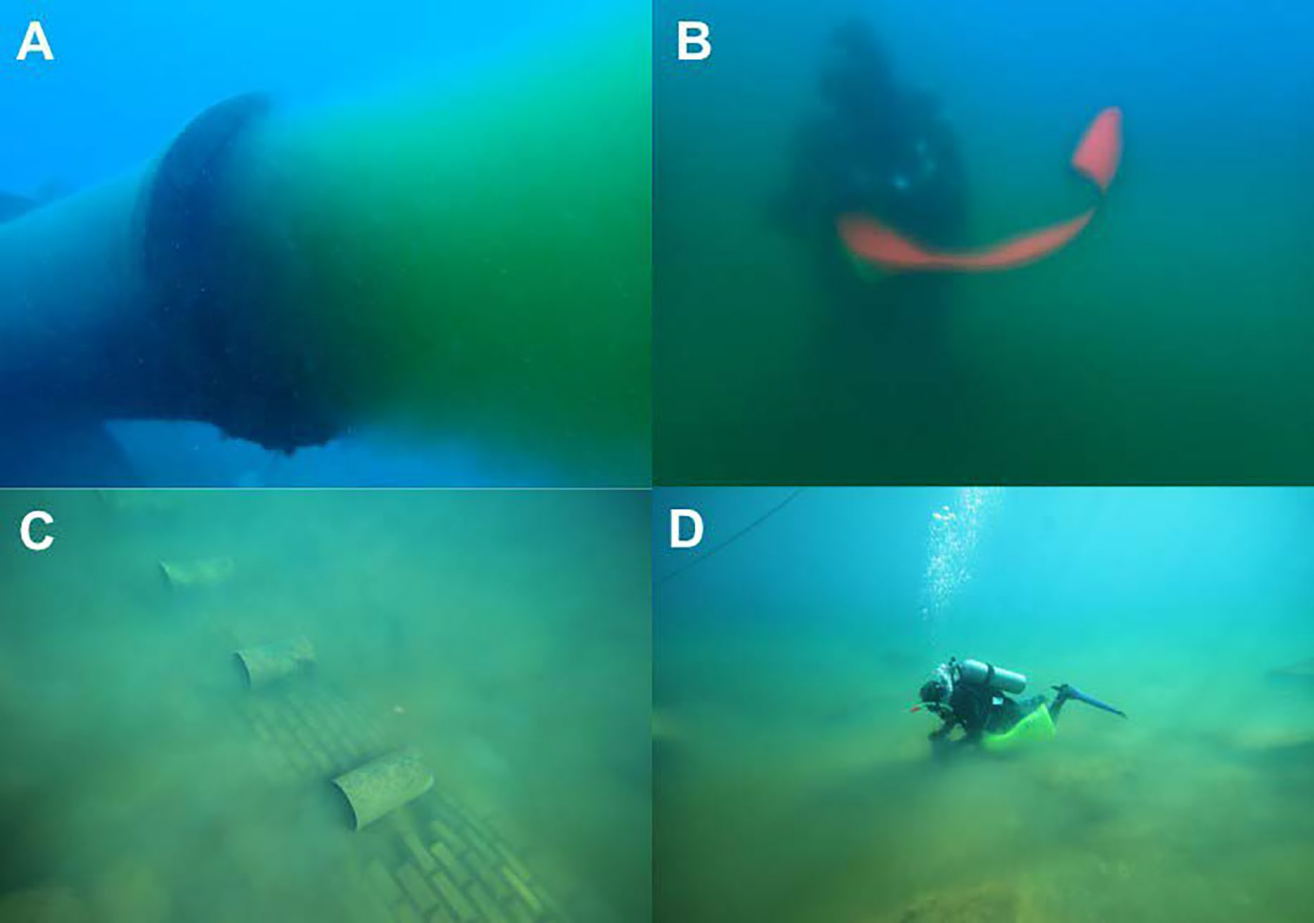
Iron emissions from desalination plants in Cyprus occur intermittently during backwashing of the plants and are, when they occur, highly visible to divers. **A**, **B** Larnaca Desalination Plant, 22 November 2021 (photos by the 1 st author). **C**, **D** Dhekelia Desalination Plant, 23 November 2023 (photos courtesy of Burak Ali Cicek, Famagusta). **D** shows the first author during his diving surveys

However, there are other environmental impacts largely eclipsed by those associated with energy provision and brine discharge, which deserve attention. Reverse osmosis requires antiscalants and coagulants for providing a clean feedstock water in order to achieve a long, economically viable and sustainable lifetime of the membranes (Belkin et al. 2017). A widely used coagulant is iron (III) chloride (FeCl_3_), which is used to remove phytoplankton and organic particles from the intake water (Belkin et al. 2017). Every couple of hours, usually during the backwashing of the system, a mixture of iron(III) and coagulated organic matter is released back into the sea via the outfall pipes of the regular brine discharge. This process is highly visible to divers conducting surveys in the outfall area (Fig. 1), with the striking appearance of a coffee-like cloud appearing together with the regular brine and accumulating in the typical brine layer on the seabed which is denser than normal seawater and takes a while to dissolve and dilute (Xevgenos et al. 2021). This is the likely origin of the increased iron concentrations encountered in seawater and sediment samples collected in Mediterranean systems, in the vicinity of the outfall areas (Grammatiki et al. 2025a, 2025b). Few studies have considered potential implications of this for the marine environment. Among these, the study by Belkin et al. (2017) is exemplary by investigating both in situ marine microbial communities near the Ashkelon Desalination Plant in Israel as well as communities in mesocosms exposed to both FeCl_3_ coagulants (and polyphosphonate-based antiscalants). It showed that FeCl_3_ immediately removed certain taxonomic groups such as Flavobacteria and Dinoflagellates, underlining its effectiveness as a coagulant removing microorganisms. The same study found that physiological changes accompanied the aforementioned changes in community composition. The predominant impact of Fe additions over 10 days appeared to be a consequence of the initial removal of the cells from the water (by aggregation and sinking), caused by the iron-hydroxides creating flocks of organic matter in the water. These processes resulted in a significant reduction in algal biomass indices (Chl a). However, taken together, this study considers the reduction in algal and microbial diversity and biomass due to the localized FeCl_3_ addition. We are not aware of any studies that have considered the implications of desalination plants adding iron as a limiting micronutrient to a marine ecosystem. It is the purpose of this review paper to elicit research interest into what should be considered an important, yet neglected aspect of marine environmental impacts of seawater desalination plants.

## Operational and engineering aspects

Desalination typically involves four stages: (1) intake and pretreatment, (2) desalination (reverse osmosis, RO), (3) post-treatment, and (4) brine disposal (Alvarado-Revilla et al. 2016). When added to seawater, FeCl₃ hydrolyzes to form ferric hydroxide, Fe(OH)₃, which aggregates impurities that can then be filtered out. This process effectively reduces membrane fouling and ensures efficient RO operation (Alvarado-Revilla et al. 2016). However, residual iron and associated compounds can remain in backwash water or enter brine discharges, potentially altering the local marine environment (Voutchkov 2011).

Typically, a Reverse Osmosis plant is fed with water that has been treated first by a pre-treatment plant aiming to protect this from fouling that can be caused by suspended solids, organics and scaling minerals (Salinas Rodriguez et al. 2021). To do so, several chemicals are used which can be grouped in the following three wider categories: (a) Coagulants; (b) flocculants and (c) antiscalants (Alvarado-Revilla et al. 2016). The focus of this paper is the discharge of ferric hydroxide (also called rust) that is related to the dosing of ferrous salts (either ferric chloride or ferric sulphate) as a coagulant. The dosing concentration of the ferrous salts depends on the feed water quality, but normally it ranges from 0.5 to 10 mg Fe/L, although in some cases doses as high as 20 mg Fe/L have been reported (Edzwald and Haarhoff 2011).

The quality of the treated water before RO is indicated by the Silt Density Index (SDI), which is a standard method used to measure the performance of pre-treatment before the RO membrane. Most membrane manufacturers recommend an SDI value of less than 3. Recent studies report (EU 2022) that as of February 2022 there were 20,971 desalination plants globally producing 97.2 million m^3^ of drinking water per day and releasing 150 million m^3^ of brine per day. Eke et al (2020) report that seawater desalination accounted for approximately 57% of the total global installed desalination capacity in 2020 (desalination of brackish water accounted for 20% and other types of source water for 23%, respectively). Assuming a similar percentage for 2022, the seawater desalination capacity can be estimated at approx. 55,404,000 m^3^/day. Assuming a recovery ratio of 40%, we can assume that approx. 141,000,000 m^3^ of seawater were treated per day globally.

**Fig. 2:**
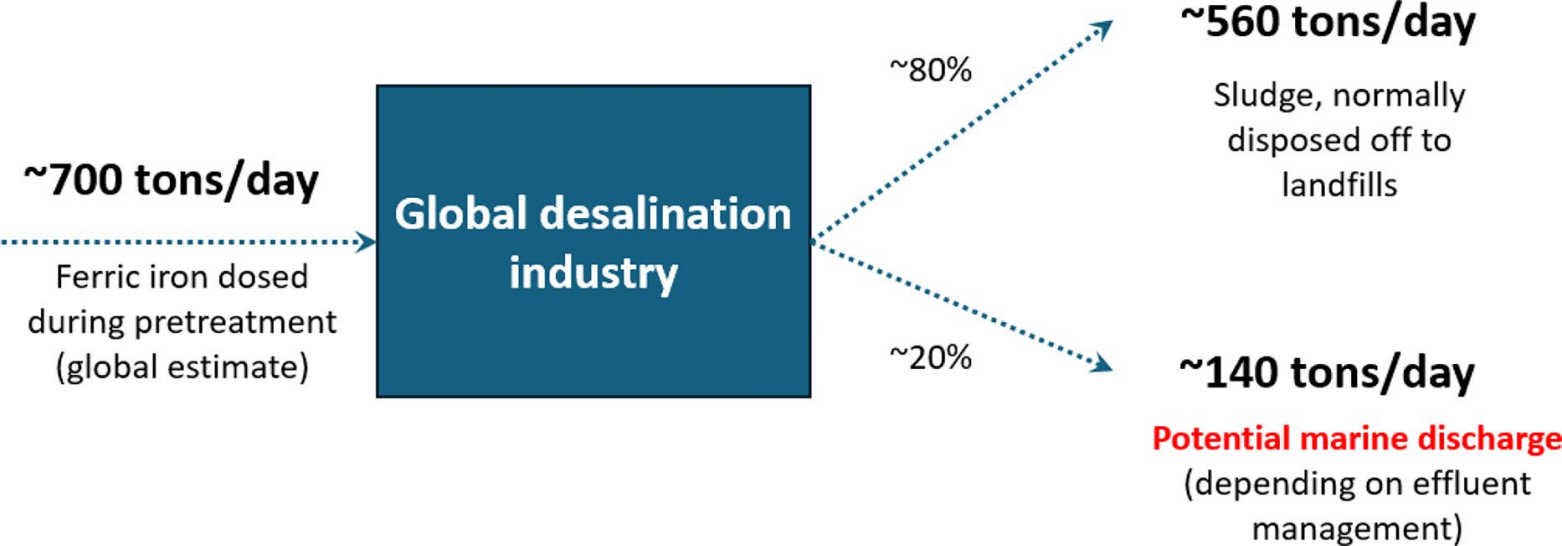
Illustrative global mass balance of iron use in desalination plants

If iron-based coagulants are used during pretreatment and a representative dosage of 5 mg Fe/L is assumed, the global ferric iron dosing associated with seawater desalination operations can be estimated at around 705 tons of iron(III) per day. Most of this iron content—in the order of ~ 80% (Voutchkov 2011)—hydrolyses quickly forming Fe(OH)_3_ and is removed together with the colloidal solids in the form of sludge during the pre-treatment and is typically managed on land. The concentration of total suspended solids in the spent backwash water is a function of the TSS concentration of the source water as well as the dosage of the applied iron coagulant and can be calculated as follows:

The remaining (~ 20%) may be discharged into the marine environment, depending on plant configuration and effluent management practices. An illustrative global mass balance of iron use in desalination plants is presented in Fig. 2.

It is noted that this calculation assumes that all desalination plants are using iron (e.g. in the form of iron chloride or iron sulphate) however, pretreatment strategies vary, and aluminum-based coagulants, organic polymers, or no coagulants may also be applied. Consequently, the presented values should be interpreted as an order-of-magnitude, upper-bound estimate rather than a precise global flux.

Regarding iron speciation after discharge, the scarce available evidence suggests that the released iron will be predominantly present as particulate or colloidal Fe(III), with only a minor fraction remaining in dissolved form, largely stabilized through organic complexation (Gledhill and Buck 2012; Shaked et al. 2025). There is clearly a need for further research here.

### Use of ferric chloride or ferric sulphate as a coagulant in RO pre-treatment

All RO membrane manufacturers’ warranties require that feedwater be treated to some minimum acceptable level (Salinas Rodriguez et al. 2021). The universal guideline for establishing RO feedwater quality is determined by its fouling or plugging tendency as determined by its Silt Density Index (SDI). The SDI is based on the timed flow of the water sample through a 0.45 μm filter at a constant pressure of 2-bar. An SDI value greater than 4.0 usually indicates that the water requires additional pretreatment.

Historically, a variety of unit processes, including conventional coagulation, flocculation, sedimentation and granular media filtration—either a single- or two-stage system with pressure or gravity filters—have been employed to remove suspended solids. However, in many surface water applications, particularly in large seawater reverse osmosis (SWRO) installations, there is a growing trend to employ dissolved air flotation (DAF) to improve the removal of algae and other low-density organic matter, followed by microfiltration (MF) or ultrafiltration (UF) to remove remaining particulates (Salinas Rodriguez et al. 2021).

**Fig. 3:**
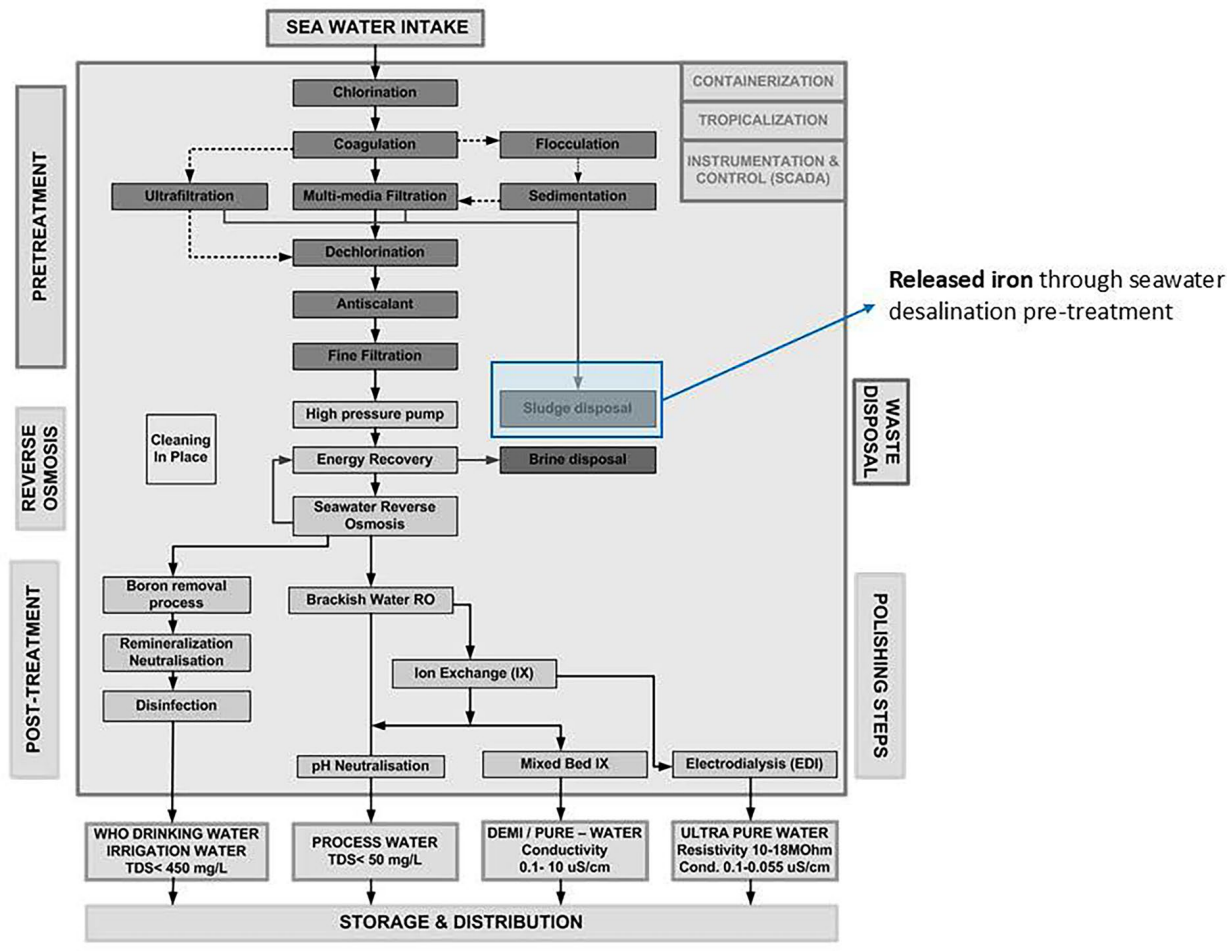
Illustration of RO pre-treatment options (sludge disposal stemming from the coagulation & flocculation step highlighted with a blue box). Reproduced with kind permission from Lenntech B.V. (Delft, Netherlands)

In applications with relatively low levels of suspended solids, MF/UF systems may be operated in a direct filtration mode, with no additional pretreatment other than an 80- to 200-micron strainer. In most cases, provisions are made to include a primary coagulant such as ferric chloride or ferric sulphate seasonally, or as conditions may warrant. An MF/UF system usually produces a filtrate with an SDI of 0.8 to 2.5. Figure 3 illustrates the available pre-treatment options in RO plants (Salinas Rodrigues et al. 2021).

## Regulatory and legal frameworks for iron(III) pollution in marine environments

### Global and regional frameworks

Building on the preceding discussion of iron(III) use and discharge in desalination processes, this section examines how regulatory and legal frameworks address iron(III) pollution in marine environments. Despite the global expansion of desalination activities and the increasing recognition of their environmental implications, iron(III) remains largely unaddressed in the dominant international and regional marine pollution frameworks. Instruments such as the United Nations Environment Programme (UNEP) Global Programme of Action (Unep 1995) and the associated Regional Seas Conventions, including the Barcelona Convention (Mediterranean), the Jeddah Convention (Red Sea), and the Kuwait Regional Convention (ROPME Sea Area), establish broad legal obligations to prevent and reduce pollution from land-based sources (Unep 2017a, 2017b). However, these frameworks remain general in scope and lack pollutant-specific emission standards for iron and related metals. This analysis highlights a persistent conceptual and regulatory gap, raising the question of how adequate existing frameworks truly are in addressing iron(III) pollution originating from desalination and coastal industries.

Iron continues to be regarded primarily as a naturally occurring micronutrient essential for biological productivity rather than as a contaminant of concern. Yet, a growing body of scientific evidence suggests that under certain anthropogenic conditions, iron(III) can become ecologically disruptive. Elevated concentrations resulting from corrosion of pipelines, coagulant discharge, and desalination brine effluents have been shown to modify sediment redox conditions, enhance eutrophication potential, and alter benthic community structure (Belkin et al. 2017; Hosseini et al. 2021). This emerging understanding underscores a growing disparity between current scientific insight and the regulatory frameworks that govern marine pollution control. The lack of explicit iron(III) standards within the UNEP and GPA frameworks demonstrates that iron has remained conceptually trapped between the categories of “nutrient” and “pollutant,” limiting its formal recognition in marine environmental law (Unep 2006).

At the national and interregional levels, approaches to iron regulation and monitoring vary widely, often reflecting historical perceptions of risk rather than empirical evidence. For instance, the U.S. Environmental Protection Agency (EPA), under the Clean Water Act (CWA), recommends 0.3 mg L⁻^1^ for marine waters and 1.0 mg L⁻^1^ for freshwater within its National Recommended Water Quality Criteria (EPA 2009). Similarly, the Australian and New Zealand Environment and Conservation Council (ANZECC-ARMCANZ 2000) identifies iron as a parameter requiring surveillance in industrial effluents and sets a trigger value of 0.3 mg L⁻^1^ for marine ecosystems. While these values are advisory rather than legally binding, they nonetheless represent precautionary thresholds that reflect an evolving understanding of metal bioavailability and ecological risk.

In contrast, the European Union’s legal framework, comprising the Water Framework Directive (EU 2000) and the Marine Strategy Framework Directive (EU 2008b), continues to exclude iron from its list of priority substances. The absence of iron(III) within the Environmental Quality Standards Directive (EU 2008a) and its amendment Directive (EU 2013a, 2013b) highlights a regulatory inertia that privileges acutely toxic heavy metals such as mercury, cadmium, and lead over metals like iron that exert chronic, sub-lethal, or biogeochemical effects. This disparity highlights an ongoing challenge: whether current regulatory thresholds and monitoring approaches are sufficient to safeguard sensitive ecosystems such as coral reefs and seagrass meadows, particularly in semi-enclosed or hypersaline systems where iron concentrations may accumulate through repeated desalination discharge. At the national level, case studies from the Eastern Mediterranean (Cyprus) and the Arabian Peninsula (Saudi Arabia) illustrate how these frameworks are applied in practice (below).

Addressing these challenges is essential to align desalination operations with global efforts toward sustainable marine pollution management.

### National frameworks: Cyprus and Saudi Arabia

Within the Mediterranean basin, Cyprus provides a useful illustration of how regional directives are translated into national regulation. As a Party to the Barcelona Convention (Cyprus 2001) and the LBS Protocol (Protocol for the Protection of the Mediterranean Sea against Pollution from Land-Based Sources; UNEP 1980), Cyprus is legally obligated to control industrial and desalination-related discharges through a permitting system. The *Water and Soil Pollution Control Law* (Cyprus 2002) applies a combined framework based on Water Quality Objectives (WQOs) and Emission Limit Values (ELVs), under which each facility must obtain a discharge permit specifying limits for particular pollutants. According to the *MSFD Cyprus Report 2017–2022* (Cyprus 2024), Cyprus operates seven coastal monitoring stations for seawater contaminants, seven stations for sediments, and 26 MEDITS offshore stations for heavy-metal assessment, measuring ten metals and PAHs using methodologies aligned with the Water Framework Directive (EU 2000) and the UNEP/MAP Integrated Monitoring and Assessment Programme (IMAP) pollution guidelines (UNEP/MAP 2017). However, no national numeric threshold values exist for iron(III). Instead, metal concentrations are benchmarked against the Priority Substances Directive (EU 2008a) and the MED POL Background Assessment Concentrations (BACs) and Environmental Assessment Criteria (EACs) under UNEP/MAP. Desalination plant operators are required to monitor iron levels in seawater and sediments (and to submit these data, together with a range of other potential pollutants, to the Cyprus Government Department of Environment, Ministry of Agriculture, Rural Development and Envitonment) at their outfall sites every 2 years for obtaining and maintaining their government-issued license to operate. While this approach ensures methodological consistency with EU requirements, it also reflects a fragmented reliance on permit-based evaluations rather than pollutant-specific regulation. Consequently, the Cypriot framework raises questions about how effectively current compliance systems capture long-term and cumulative trace-metal effects, particularly in light of the country’s high dependence on desalination as a freshwater source (DFMR 2024).

In contrast, Saudi Arabia represents one of the few countries to adopt a pollutant-specific and legally binding regulatory approach to iron(III). The Environmental Law (KSA 2020) and its Executive Regulations for the Protection of Aqueous Media from Pollution (DFMR 2024) establish explicit discharge thresholds for iron(III): 0.1 mg/L for ecologically sensitive areas, 0.5 mg/L for general coastal waters, and 1.0 mg/L for industrial mixing zones (≤ 100 m) (KSA 2020, 2021b). Complementary instruments, including the Executive Regulation for Marine and Coastal Management (KSA 2021a) and the Environmental Guideline for Desalination Plants (NCEC 2021a), require operators to implement Environmental Impact Assessments (EIAs), effluent modeling, and continuous monitoring (KSA 2021a, NCEC 2021b). This tiered and enforceable framework marks a shift from principle-based governance to a performance-oriented model that directly links regulatory thresholds with ecological vulnerability. Van der Merwe et al. provide a review of seawater desalination-related environmental governance for Saudi Arabia (2013).

Nevertheless, while Saudi Arabia’s regulatory system provides a model of precision and accountability, implementation challenges remain. Ensuring inter-agency coordination, maintaining data transparency, and incorporating emerging analytical technologies such as eDNA metabarcoding or sensor-based monitoring will be critical for sustaining long-term compliance both for desalination brine discharge in general and specifically for iron. These issues point toward a broader need for policy innovation and institutional capacity-building to ensure that iron(III) regulation evolves alongside advances in marine pollution science.

## Regional case studies

### Cyprus

Given the location of Cyprus in the eastern Mediterranean which is a region that is naturally subject to large amounts of iron-rich aeolian dust deposition, the impact of iron(III) from seawater desalination plants in Cyprus on the island’s shallow-water environment may be negligible. The same applies certainly also for the Gulf Region, where the effects massive amounts of aeolian dust from surrounding deserts and the riverine input of sediments by the Euphrates and Tigris rivers (Sheppard et al. 2010) compounded by natural and anthropogenic temperature and salinity stress (Hasan et al. 2023) are likely to far outweigh the emissions of iron from the region’s many large desalination plants, constituting the largest concentration of desalination capacity in the world. However, things may be different in places which are remote from areas with large amounts of riverine or aeolian iron input, such as small islands in the equatorial and southern Pacific, and which are considered HNLC regions (Timermans et al. 1997).

The Mediterranean Sea is, for the most part, an oligotrophic, or nutrient-poor, marine system (Heimbürger et al. 2014). Within this system, Cyprus is situated in the ultra-oligotrophic Levantine Basin (Herut et al. 1999). This region is uniquely characterized by its Low Nutrient Low Chlorophyll (LNLC) status (Astrahan et al. 2016). In such environments, the supply of nutrients from external sources is a primary factor controlling biological productivity. The Cypriot marine environment is subject to episodic, high-magnitude inputs of Saharan dust, particularly in spring and autumn (Herut et al. 1999, 2002, 2005; Herut 2005). This dust is an important source of limiting nutrients (N, P) and crustal-derived trace metals, including iron (Herut et al. 1999, 2002, 2005; Herut 2005). Guerzoni et al. (1999) highlighted that atmospheric deposition in the Mediterranean is a fundamental driver of biogeochemistry, suggesting that phytoplankton blooms can be triggered by the deposition of N, P, or Fe. This input is important for fueling the microbial loop, a defining characteristic of nutrient cycling in the ultra-oligotrophic Levantine Basin. Guieu et al. (2010) estimated that at the scale of the entire Mediterranean Sea, the atmospheric iron deposition of is ~ 89% lithogenic in origin.

Recent studies in this context became possible because of an intensive sampling program within the framework of the EU project WATER-MINING, exploring the impacts of brine discharge from 2 seawater desalination plants in Larnaca and Dhekelia (Xevgenos et al. 2021). Sampling surveys conducted between May 2022 and February 2023 assessed sediment quality near the Larnaca and Dhekelia desalination plant outfalls relative to reference stations 1.5 km away (Grammatiki et al. 2025a). Both plants have a production capacity of 60,000 m^3^/day (Xevgenos et al. 2016). At Larnaca, where the seabed is composed of fine sand, water-soluble iron concentrations reached 4.3 mg/kg at 50 m from the discharge, compared to a background value of 2.4 mg/kg. At Dhekelia, characterized by coarse sand, iron levels were lower overall, rising from 0.5 mg/kg at the reference site to 1.1 mg/kg at 150 m. These results coincide with underwater visual observations of turbidity and “red brine”, indicating potential inputs from pipeline corrosion or coagulants used in the plant’s operation (Grammatiki et al 2025a, b). Overall, this constituted a hitherto almost unparalleled research effort of several aspects of the impacts of desalination brine discharge upon the biodiversity and other aspects of the coastal benthic marine environment of Cyprus which is comparatively less studied than the western Mediterranean, resulting in a detailed investigation of sediment-dwelling microinvertebrates (Grammatiki et al. 2025a) and diatoms (Grammatiki et al. 2025b), but also unexpected new records of Polychaetes (Rousou et al. 2023), the description of 3 new Crustacean species (Stepien et al. 2024), and the first record of a pathogen infecting microphytobenthos from the Mediterranean (Scholz et al. 2025).

### Baja California, Mexico, and California, USA

Baja California is facing an unprecedented water crisis, exacerbated by an 18% cut in the Colorado River’s water allocation (equivalent to 346 million cubic meters) implemented in 2025 (Guerra 2025). This situation has driven the accelerated development of desalination infrastructure in the region, positioning desalination plants as a strategic solution to reduce dependence on the Colorado River and to ensure water security in the border region (Sánchez Munguía 2020).

The Pacific coast of Baja California and southern California is part of an upwelling-influenced coast that supports extensive kelp forests dominated by the giant kelp *Macrocystis pyrifera* (Schiel and Foster 2015). The discharge of concentrated brine, an inevitable byproduct of desalination, poses a potential threat to these organisms.

In the absence of local studies, evidence must be drawn from analogous systems elsewhere. A six-year M-BACI study in Australia documented that the construction of desalination outlets caused a decrease in *Ecklonia radiata* (kelp) coverage and an increase in algal mat coverage up to 55 m from the outlet (Kelaher and Coleman 2022), and transplant experiments with the brown alga *Dictyota* near brine discharges in northern Chile and the Spanish Mediterranean reported marked photosynthetic and oxidative-stress responses (Muñoz et al. 2023). Both lines of evidence concern the hypersaline brine plume rather than the iron(III) coagulant co-discharged from the same outlet, whose potential as a trace element input to these macrophyte-dominated communities remains unexplored.

Smaller-scale plants operate in Baja California, mainly concentrated on the Pacific coast. The plant in Ensenada (Baja California), which began operating in 2018, was the first large-scale plant on Mexico’s northwest Pacific coast (Sánchez Munguía 2020). There are also smaller plants on Isla de Cedros (5 L/second) and multiple facilities in the San Quintín Valley dedicated mainly to export agriculture, with at least 11 brine discharge points reported in San Quintín Bay (Guerrero and Valenzuela 2023). Also in Baja California, the most ambitious project is the Rosarito Desalination Plant (2200 L/second, equivalent to 50 million gallons per day), with an estimated commencement of operation by the end of 2027 (Aragon 2024). This project has an EIA submitted to Mexico’s Ministry of Environment and Natural Resources since 2014 (SEMARNAT 2014) and seeks to reduce dependence on water from the Colorado River (SEMARNAT 2014). According to Mexico’s National Water Commission (CONAGUA) reports, there are more than 400 desalination plants in operation throughout Mexico, although most are small-scale private facilities (Godoy 2021; Quinto 2024).

The most comprehensive scientific study in the region relates to the Carlsbad plant in California. The findings revealed that the brine discharge plume extended approximately 600 m offshore with salinity up to 2.7 units above ambient (Lykkebo Petersen et al. 2019). No significant changes in benthic organisms were detected specific to this plant, which is attributed to the long history of previous anthropogenic impacts in the area (Lykkebo Petersen et al. 2019). However, neither this study nor the regulatory assessments carried out for the Mexican facilities considered the iron discharged with the coagulant. Indeed, the San Quintín Valley, the first large-scale plant in Ensenada, and the Rosarito project have all been subject to regulatory review (Cooley et al. 2006; Sánchez Munguía 2020; SEMARNAT 2014), but none has a published study on its iron emissions.

The expansion of desalination capacity constitutes a viable strategy for addressing the regional water crisis both in Mexico and the USA, and the regulatory environmental impact assessment process, such as the EIA for the Rosarito project (SEMARNAT 2014), is an important step. However, compliance with these requirements does not replace the need for independent and published scientific research. It is recommended to supplement regulatory assessments with scientific monitoring programs using the M-BACI design, to publish results in peer-reviewed scientific journals (Cooley et al. 2006, who recommend characterization for at least one year prior to construction), to develop specific research on macroalgae and seagrass species in Baja California, and to establish binational Mexico-US standards for scientific monitoring. In this sense, such monitoring should also include the iron(III) discharged with the coagulant, in particular its speciation, fate, and bioavailability.

Like elsewhere in the world, the desalination plants expanding along this coast use ferric chloride as a coagulant during pre-treatment, as described above, and therefore release iron(III) into the marine environment. In this sense, the California-Baja California region is a clear example of the problem addressed in this review: monitoring and regulation have focused almost exclusively on salinity, while the iron emitted by these plants and its possible effects on the kelp forests and seagrass beds of the region remain unstudied.

### Northern Chile

Antofagasta, located on the northern coast of Chile and adjacent to the Atacama Desert, is one of the driest regions in the world (Clarke 2006). The region faces extreme aridity, with very limited freshwater resources and increasing demand from both the mining industry and a growing urban population (Clarke 2006). Consequently, reliance on conventional freshwater sources is not sustainable. Seawater desalination has therefore become essential for securing water supplies for domestic and industrial use. Antofagasta’s successful adoption of desalination marks a major achievement in water management for arid regions. The city’s main desalination facility, the Planta Desaladora Norte (formerly “La Chimba”), began operation around 2003 and expanded its capacity from approximately 650 L/s to 1,071 L/s by 2020 (Burk et al. 2013; Herrera-León et al. 2019). As of 2025, Antofagasta is reported to rely almost entirely on desalinated seawater for drinking water supply, making it one of the first cities in Latin America to achieve complete desalination dependence (Silva 2025).

In Antofagasta, RO plants typically achieve freshwater recovery ratios of ~ 0.42 (Silva and Purcell 2024). Given the high energy requirements of RO and the region’s costly electricity, desalination represents both technological and economic challenges. In addition, environmental concerns arise. In the Antofagasta plants, like in most RO-based desalination plants worldwide, ferric chloride (FeCl_3_), is applied as a coagulant in desalination pretreatment to remove suspended particles, colloids, and organic matter (Ruffino et al. 2022).

## Conclusions

To the best of our knowledge, this constitutes the first paper to consider the potential importance of iron emissions from seawater desalination plants for the marine environment. This review establishes a strong case that the discharge of iron(III) from seawater desalination plants represents a previously overlooked, yet potentially significant, anthropogenic flux of this essential micronutrient into coastal marine ecosystems. Our analysis, which combines a global mass balance estimate of iron use in desalination with regional case studies, reveals that these operations release substantial amounts of iron, predominantly as particulate or colloidal ferric hydroxide. While this source may be dwarfed by natural inputs like aeolian dust in regions such as the Mediterranean or the Gulf, it could be critically important in more oligotrophic or HNLC coastal waters where iron is a primary limiting factor. The intermittent nature of these discharges during backwashing, coupled with their high visibility and potential to alter local biogeochemistry, strengthens the need for a change on how we view the environmental impact of desalination with regards to impacts that go beyond energy consumption and brine salinity.

This review highlights a substantial disconnect between current scientific understanding and existing regulatory frameworks. While iron is recognised as a vital nutrient, emerging evidence from coastal studies demonstrates that anthropogenic enrichment can induce ecological shifts, modify sediment redox conditions, and even favour harmful algal bloom species. The lack of specific iron(III) emission standards in major international conventions and EU directives (in contrast for example with the enforceable, tiered approach adopted by Saudi Arabia) illustrates a global regulatory gap. This gap leaves potentially vulnerable ecosystems, such as those in Cyprus or the ecologically critical kelp forests and seagrass beds of Baja California, without adequate protection from the chronic, sub-lethal effects of iron enrichment. The very nature of iron— being both a “nutrient” and a “pollutant”—has hampered its formal recognition in marine environmental law, making it imperative that future policy evolves to incorporate the new scientific realities presented here. The research presented here is a first step, and it is our hope that this paper will initiate thinking that will lead into future studies focusing on in-situ monitoring of iron speciation and bioavailability in desalination outfalls, as well as long-term ecological surveys of benthic and pelagic communities. Understanding the interplay between desalination-derived iron, natural iron cycles, and the complex biological strategies for iron uptake, will be crucial.

## Data Availability

No datasets were generated or analysed during the current study.

## Abbreviations

DAF: Dissolved air flotation
MF: Microfiltration
RO: Reverse osmosis
UF: Ultrafiltration

## References

[CR1] Alvarado-Revilla F, Brown H, Charamidi M, Elkins I, Filou E, Gasson C, Uzelac J (2016) Desalination markets 2016. Media Analytics Ltd, Oxford

[CR2] Amin SA, Küpper FC, Green DH, Harris WR, Carrano CJ (2007) Boron binding by a siderophore isolated from marine bacteria associated with the toxic dinoflagellate *Gymnodinium catenatum*. J Am Chem Soc 129:478–479 17226996 10.1021/ja067369u

[CR3] Amin SA, Green DH, Küpper FC, Carrano CJ (2009) Vibrioferrin, an unusual marine siderophore: iron binding, photochemistry, and biological implications. Inorg Chem 48:11451–11458 19821595 10.1021/ic9016883

[CR4] Anzecc-Armcanz (2000) Australian and New Zealand Guidelines for fresh and marine water quality. In: (ARMCANZ), ANZECC & ARMCANZ. (ed.). Canberra

[CR5] Aragon J (2024) Desaladora de Rosarito podría estar lista a finales de 2027: Seproa. Semanario ZETA

[CR6] Ardyna M, Lacour L, Sergi S, D’ovidio F, Sallée J-B, Rembauville M, Blain S, Tagliabue A, Schlitzer R, Jeandel C, Arrigo KR, Claustre H (2019) Hydrothermal vents trigger massive phytoplankton blooms in the Southern Ocean. Nat Commun 10:2451 31165724 10.1038/s41467-019-09973-6 PMC6549147

[CR7] Armbrust EV, Berges JA, Green BR, Martinez D, Putnam NH, Zhou S, Allen AE, Apt KE, Bechner M, Brzezinski MA, Chaal BK, Chiovitti A, Davis AK, Demarest MS, Detter JC, Glavina T, Goodstein D, Hadi MZ, Hellsten U, Hildebrand M, Jenkins BD, Jurka J, Kapitonov VV, Kröger N, Lau WWY, Lane TW, Larimer FW, Lippmeier JC, Lucas S, Medina M, Montsant A, Obornik M, Schnitzler Parker M, Palenik B, Pazour GJ, Richardson PM, Rynearson TA, Saito MA, Schwartz DC, Thamatrakoln K, Valentin K, Vardi A, Wilkerson FP, Rokhsar DS (2004) The genome of the diatom *Thalassiosira pseudonana*: ecology, evolution, and metabolism. Science 306:79–86 15459382 10.1126/science.1101156

[CR8] Astrahan P, Herut B, Paytan A, Rahav E (2016) The impact of dry atmospheric deposition on the sea-surface microlayer in the SE Mediterranean Sea: an experimental approach. Front Mar Sci 3:222

[CR9] Avramidi E, Gomez Garcia S, Papaspyrou S, Louca V, Xevgenos D, Küpper FC (2022) Benthic biodiversity at the brine discharge sites in the Port of Rotterdam. Water Resources Industry 27:100173

[CR10] Baar H, Buma AGJ, Nolting RF, Cadee GC, Jacques G, Treguer PJ (1990) On iron limitation of the Southern Ocean: experimental observations in the Weddell and Scotia Seas. Mar Ecol Prog Ser 65:105–122

[CR11] Barbeau K (2006) Photochemistry of organic iron(III) complexing ligands in oceanic systems. Photochem Photobiol 82:1505–1516 16968114 10.1562/2006-06-16-IR-935

[CR12] Barbeau K, Rue EL, Bruland KW, Butler A (2001) Photochemical cycling of iron in the surface ocean mediated by microbial iron(III)-binding ligands. Nature 413:409–413 11574885 10.1038/35096545

[CR13] Barbeau K, Zhang GP, Live DH, Butler A (2002) Petrobactin, a photoreactive siderophore produced by the oil-degrading marine bacterium *Marinobacter hydrocarbonoclasticus*. J Am Chem Soc 124:378–379 11792199 10.1021/ja0119088

[CR14] Barbeau K, Rue EL, Trick CG, Bruland KT, Butler A (2003) Photochemical reactivity of siderophores produced by marine heterotrophic bacteria and cyanobacteria based on characteristic Fe(III) binding groups. Limnol Oceanogr 48:1069–1078

[CR15] Belkin N, Rahav E, Elifantz H, Kress N, Berman-Frank I (2017) The effect of coagulants and antiscalants discharged with seawater desalination brines on coastal microbial communities: a laboratory and in situ study from the southeastern Mediterranean. Water Res 110:321–331 28063294 10.1016/j.watres.2016.12.013

[CR145] Böttger LH, Miller, EP, Andresen C, Matzanke BF, Küpper FC, Carrano CJ (2012) Atypical iron storage in marine brown algae: a multidisciplinary study of iron transport and storage in *Ectocarpus siliculosus*. J Exp Bot 63(16):5763–5772 10.1093/jxb/ers225 PMC3467295 22945940

[CR16] Bowler C, Allen AE, Badger JH, Grimwood J, Jabbari K, Kuo A, Maheswari U, Martens C, Maumus F, Otillar RP, Rayko E, Salamov A, Vandepoele K, Beszteri B, Gruber A, Heijde M, Katinka M, Mock T, Valentin K, Verret F, Berges JA, Brownlee C, Cadoret JP, Chiovitti A, Choi CJ, Coesel S, De Martino A, Detter JC, Durkin C, Falciatore A, Fournet J, Haruta M, Huysman MJJ, Jenkins BD, Jiroutova K, Jorgensen RE, Joubert Y, Kaplan A, Kroger N, Kroth PG, La Roche J, Lindquist E, Lommer M, Martin-Jezequel V, Lopez PJ, Lucas S, Mangogna M, Mcginnis K, Medlin LK, Montsant A, Oudot-Le Secq MP, Napoli C, Obornik M, Parker MS, Petit JL, Porcel BM, Poulsen N, Robison M, Rychlewski L, Rynearson TA, Schmutz J, Shapiro H, Siaut M, Stanley M, Sussman MR, Taylor AR, Vardi A, Von Dassow P, Vyverman W, Willis A, Wyrwicz LS, Rokhsar DS, Weissenbach J, Armbrust EV, Green BR, Van De Peer Y, Grigoriev IV (2008) The *Phaeodactylum* genome reveals the evolutionary history of diatom genomes. Nature 456:239–244 18923393 10.1038/nature07410

[CR17] Boyd PW, Muggli DL, Varela DE, Goldblatt RH, Chretien R, Orians KJ, Harrison PJ (1996) In vitro iron enrichment experiments in the NE subarctic Pacific. Mar Ecol Prog Ser 136:179–193

[CR18] Boyd PW, Watson AJ, Law CS, Abraham ER, Trull T, Murdoch R, Bakker DCE, Bowie AR, Buesseler KO, Chang H, Charette M, Croot P, Downing K, Frew R, Gall M, Hadfield M, Hall J, Harvey M, Jameson G, Laroche J, Liddicoat M, Ling R, Maldonado MT, Mckay RM, Nodder S, Pickmere S, Pridmore R, Rintoul S, Safi K, Sutton P, Strzepek R, Tanneberger K, Turner S, Waite A, Zeldis J (2000) A mesoscale phytoplankton bloom in the polar Southern Ocean stimulated by iron fertilization. Nature 407:695–702 11048709 10.1038/35037500

[CR19] Boyd PW, Ellwood MJ, Tagliabue A, Twining BS (2017) Biotic and abiotic retention, recycling and remineralization of metals in the ocean. Nat Geosci 10:167–173

[CR20] Briat JF, Duc C, Ravet K, Gaymard F (2010) Ferritins and iron storage in plants. Biochim Biophys Acta 1800:806–814 20026187 10.1016/j.bbagen.2009.12.003

[CR21] Browning TJ, Achterberg EP, Engel A, Mawji E (2021) Manganese co-limitation of phytoplankton growth and major nutrient drawdown in the Southern Ocean. Nat Commun 12:884 33563991 10.1038/s41467-021-21122-6 PMC7873070

[CR22] Burk RL, Dixon MB, Kim-Hak D and Martiz Vega P (2013) A comparison of three reverse osmosis membranes at La Chimba desalination plant, Antofagasta, Chile. Proceedings of the International Desalination Association World Congress on Desalination and Water Reuse. Tianjin, China, 20–25 October 2013.

[CR23] Caputi L, Carradec Q, Eveillard D, Kirilovsky A, Pelletier E, Pierella Karlusich JJ, Rocha Jimenez Vieira F, Villar E, Chaffron S, Malviya S, Scalco E, Acinas SG, Alberti A, Aury J-M, Benoiston A-S, Bertrand A, Biard T, Bittner L, Boccara M, Brum JR, Brunet C, Busseni G, Carratalà A, Claustre H, Coelho LP, Colin S, D’aniello S, Da Silva C, Del Core M, Doré H, Gasparini S, Kokoszka F, Jamet J-L, Lejeusne C, Lepoivre C, Lescot M, Lima-Mendez G, Lombard F, Lukeš J, Maillet N, Madoui M-A, Martinez E, Mazzocchi MG, Néou MB, Paz-Yepes J, Poulain J, Ramondenc S, Romagnan J-B, Roux S, Salvagio Manta D, Sanges R, Speich S, Sprovieri M, Sunagawa S, Taillandier V, Tanaka A, Tirichine L, Trottier C, Uitz J, Veluchamy A, Veselá J, Vincent F, Yau S, Kandels-Lewis S, Searson S, Dimier C, Picheral M, Coordinators TO, Bork P, Boss E, De Vargas C, Follows MJ, Grimsley N, Guidi L, Hingamp P, Karsenti E, Sordino P, Stemmann L, Sullivan MB, Tagliabue A, Zingone A, Garczarek L, D’ortenzio F, Testor P, Not F, D’alcalà MR, Wincker P, Bowler C, Iudicone D (2019) Community-level responses to iron availability in open Ocean Plankton Ecosystems. Global Biogeochem Cycles 33:391–419

[CR24] Carrano CJ, Schellenberg S, Amin SA, Green DH, Küpper FC (2009) Boron and marine life: a new look at an enigmatic bioelement. Mar Biotechnol 11:431–440 10.1007/s10126-009-9191-4 19424754

[CR25] Cary SC (2001) Response of marine bacterial community composition to iron additions in three iron-limited regimes. Limnol Oceanogr 46:1535–1545

[CR26] Cbd (2009) Scientific synthesis of the impacts of ocean fertilization on marine biodiversity. Secretariat of the Convention on Biological Diversity, Montreal

[CR27] Clarke JDA (2006) Antiquity of aridity in the Chilean Atacama Desert. Geomorphology 73:101–114

[CR28] Coale KH, Johnson KS, Fitzwater SE, Gordon RM, Tanner S, Chavez FP, Ferioli L, Sakamoto C, Rogers P, Millero F, Steinberg P, Nightingale P, Cooper D, Cochlan WP, Landry MR, Constantinou J, Rollwagen G, Trasvina A, Kudela R (1996) A massive phytoplankton bloom induced by an ecosystem-scale iron fertilization experiment in the equatorial Pacific Ocean. Nature 383:495–501 18680864 10.1038/383495a0

[CR29] Cock JM, Sterck L, Rouzé P, Scornet D, Allen AE, Amoutzias G, Anthouard V, Artiguenave F, Aury J-M, Badger JH, Beszteri B, Billiau K, Bonnet E, Bothwell JHF, Bowler C, Boyen C, Brownlee C, Carrano CJ, Charrier B, Cho GY, Coelho SM, Collén J, Corre E, Da Silva C, Delage L, Delaroque N, Dittami SM, Doulbeau S, Elias M, Farnham G, Gachon CMM, Gschloessl B, Heesch S, Jabbari K, Jubin C, Kawai H, Kimura K, Kloareg B, Küpper FC, Lang D, Le Bail A, Leblanc C, Lerouge P, Lohr M, Lopez PJ, Martens C, Maumus F, Michel G, Miranda-Saavedra D, Morales J, Moreau H, Motomura T, Nagasato C, Napoli CA, Nelson DR, Nyvall-Collén P, Peters AF, Pommier C, Potin P, Poulain P, Quesneville H, Read B, Rensing SA, Ritter A, Rousvoal S, Samanta M, Samson G, Schroeder DC, Ségurens B, Strittmatter M, Tonon T, Tregear J, Valentin K, Von Dassow P, Yamagishi T, Van De Peer Y, Wincker P (2010) The *Ectocarpus* genome and the independent evolution of multicellularity in the brown algae. Nature 465:617–621 20520714 10.1038/nature09016

[CR30] Cock JM, Godfroy O, Macaisne N, Peters AF, Coelho SM (2014) Evolution and regulation of complex life cycles: a brown algal perspective. Curr Opin Plant Biol 17:1–6 24507487 10.1016/j.pbi.2013.09.004

[CR31] Cock JM, Sterck L, Ahmed S, Allen AE, Amoutzias G, Anthouard V, Artiguenave F, Arun A, Aury JM, Badger JH, Beszteri B, Billiau K, Bonnet E, Bothwell JH, Bowler C, Boyen C, Brownlee C, Carrano CJ, Charrier B, Cho GY, Coelho SM, Collen J, Le Corguille G, Corre E, Dartevelle L, Da Silva C, Delage L, Delaroque N, Dittami SM, Doulbeau S, Elias M, Farnham G, Gachon CMM, Godfroy O, Gschloessl B, Heesch S, Jabbari K, Jubin C, Kawai H, Kimura K, Kloareg B, Küpper FC, Lang D, Le Bail A, Luthringer R, Leblanc C, Lerouge P, Lohr M, Lopez PJ, Macaisne N, Martens C, Maumus F, Michel G, Miranda-Saavedra D, Morales J, Moreau H, Motomura T, Nagasato C, Napoli CA, Nelson DR, Nyvall-Collen P, Peters AF, Pommier C, Potin P, Poulain J, Quesneville H, Read B, Rensing SA, Ritter A, Rousvoal S, Samanta M, Samson G, Schroeder DC, Scornet D, Segurens B, Strittmatter M, Tonon T, Tregear JW, Valentin K, Von Dassow P, Yamagishi T, Rouze P, Van De Peer Y, Wincker P, Ectocarpus Genome C (2012) The ectocarpus genome and brown algal genomics the ectocarpus genome consortium. In: Piganeau G (ed) Genomic insights into the biology of algae. Academic Press Ltd-Elsevier Science Ltd, London

[CR147] Cooley H, Gleick PH, Wolff G (2006) Desalination, with a grain of salt: A California perspective. Oakland, CA, Pacific Institute

[CR32] Cooper DJ, Watson AJ, Nightingale PD (1996) Large decrease in ocean-surface CO_2_ fugacity in response to in situ iron fertilization. Nature 383:511–513

[CR33] Cornejo PK, Santana MVE, Hokanson DR, Mihelcic JR, Zhang Q (2014) Carbon footprint of water reuse and desalination: a review of greenhouse gas emissions and estimation tools. J Water Reuse Desalin 4:238–252

[CR34] Crichton RR (2018) Biological inorganic chemistry, a new introduction to molecular structure and function. Elsevier/Academic Press, Amsterdam

[CR35] Cropp RA, Gabric AJ, Levasseur M, Mctainsh GH, Bowie A, Hassler CS, Law CS, Mcgowan H, Tindale N, Rossel RV (2013) The likelihood of observing dust-stimulated phytoplankton growth in waters proximal to the Australian continent. J Mar Syst 117:43–52

[CR36] Cruz-López R, Carrano CJ (2023) Iron uptake, transport and storage in marine brown algae. Biometals 36:371–383 36930341 10.1007/s10534-023-00489-7

[CR37] Cyprus RO (2001) Law 20(III)/2001 Ratifying the Barcelona Convention. In: Department of environment, M.O.A., Rural development and environment (ed.) Law 20(III)/2001 Nicosia

[CR38] Cyprus RO (2002) Water and soil pollution control law 106(I)/2002. In: Department of environment, M.O.A., Rural development and environment (ed.) Law 106(I)/2002. Nicosia

[CR39] Cyprus RO (2024) MSFD Implementation Report 2017–2022. In: Department of environment, M.O.A., Rural development and environment (ed.). Nicosia

[CR40] De Baar HJW, Boyd PW, Coale KH, Landry MR, Tsuda A, Assmy P, Bakker DCE, Bozec Y, Barber RT, Brzezinski MA, Buesseler KO, Boyé M, Croot PL, Gervais F, Gorbunov MY, Harrison PJ, Hiscock WT, Laan P, Lancelot C, Law CS, Levasseur M, Marchetti A, Millero FJ, Nishioka J, Nojiri Y, Van Oijen T, Riebesell U, Rijkenberg MJA, Saito H, Takeda S, Timmermans KR, Veldhuis MJW, Waite AM, Wong C-S (2005) Synthesis of iron fertilization experiments: From the iron age in the age of enlightenment. J Geophys Res: Oceans. 10.1029/2004JC002601

[CR41] De Los R-E, Contreras A, Lara G, Latsague M, Esse C (2020) Relations between optical properties, dinoflagellates and zooplanktonic crustaceans in Antofagasta Bay (23°S, Chile). Crustaceana 93:41–49

[CR42] Dfmr (2024) Implementation of the Marine strategy framework directive (MSFD) 2008/56/EC in the Republic of Cyprus — Articles 8, 9, and 10 (2017–2022). . Nicosia: Department of fisheries and marine research (DFMR), Ministry of agriculture, rural development and environment, Republic of Cyprus

[CR43] Edzwald JK, Haarhoff J (2011) Seawater pretreatment for reverse osmosis: chemistry, contaminants, and coagulation. Water Res 45:5428–5440 21907384 10.1016/j.watres.2011.08.014

[CR44] Eke J, Yusuf A, Giwa A, Sodiq A (2020) The global status of desalination: an assessment of current desalination technologies, plants and capacity. Desalination 495:114633

[CR45] EPA (2009) National recommended water quality criteria: aquatic life criteria table. In: Office of water, U. S. E. P. A. E. (ed.). Washington, D.C.

[CR46] EPA U (2011) Exhaust gas scrubber washwater effluent In: Agency, U. S. E. P. (ed.) EPA-842-R-11–001

[CR47] EU (2008a) Directive 2008/105/EC on environmental quality standards in the field of water policy. Official Journal of the European Union. In: Europe, E. P. A. T. C. O. (ed.) 2008/105/EC

[CR48] EU (2008b) Marine strategy framework directive. In: Europe, E. P. A. T. C. O. (ed.) 2008/56/EC

[CR49] EU (2013a) Directive 2013/39/EU amending Directives 2000/60/EC and 2008/105/EC as regards priority substances. Official Journal of the European Union. In: Europe, E. P. A. T. C. O. (ed.) 2013/39/EU

[CR50] EU (2013b) Environmental quality standards directive-amendment directive. In: Europe, E. P. A. T. C. O. (ed.) 2013/39/EU

[CR51] EU (2000) Water framework directive (WFD) 2000/60/EC: Directive 2000/60/EC of the European parliament and of the council of 23 October 2000 establishing a framework for community action in the field of water policy. In: Europe, E. P. A. C. O. (ed.) 2000/60/EC. Brussels

[CR52] EU (2022) Desalination. Available: https://blue-economy-observatory.ec.europa.eu/eu-blue-economy-sectors/desalination_en#6 [Accessed].

[CR53] Ford GC, Harrison PM, Rice JMA, Smith A, Treffry JL, White JL, Yariv J (1984) Ferritin: design and formation of an iron-storage molecule. Philos Trans R Soc Lond B Biol Sci 304:551–565 6142491 10.1098/rstb.1984.0046

[CR54] Gledhill M, Buck KN (2012) The organic complexation of iron in the marine environment: a review. Frontiers Microbiol 3:69 10.3389/fmicb.2012.00069 PMC3289268 22403574

[CR55] Godoy E (2021) México quiere beber agua del mar, pero tiene costos ambientales. IPS - Inter Press Service

[CR56] Grammatiki K, De Jonge N, Nielsen JL, García-Gomez SC, Avramidi E, Lymperaki MM, Marcou M, Ioannou G, Papatheodoulou M, Dargent O, Xevgenos D, Hesselsøe M, Küpper FC (2025a) eDNA metabarcoding of marine invertebrate communities at RO desalination plant outfalls in Cyprus. Mar Pollut Bull 214:117609 40068430 10.1016/j.marpolbul.2025.117609

[CR57] Grammatiki K, De Jonge N, Nielsen JL, Scholz B, Avramidi E, Lymperaki M, Hesselsøe M, Xevgenos D, Küpper FC (2025b) Environmental impact of brine from desalination plants on marine benthic diatom diversity. Mar Environ Res 210:107207 40483798 10.1016/j.marenvres.2025.107207

[CR58] Guerra JP (2025) Desglosan el avance de las obras de infraestructura hídrica en Baja California. La Jornada Baja California

[CR59] Guerrero C and Valenzuela A (2023) Desalination plants and their potential effect on the environment. Terra Peninsular. Available: https://terrapeninsular.org/en/desalination-plants-and-their-potential-effect-on-the-environment/

[CR60] Guerzoni S, Chester R, Dulac F, Herut B, Loÿe-Pilot M-D, Measures C, Migon C, Molinaroli E, Moulin C, Rossini P, Saydam C, Soudine A, Ziveri P (1999) The role of atmospheric deposition in the biogeochemistry of the Mediterranean Sea. Prog Oceanogr 44:147–190

[CR61] Guieu C, Loÿe-Pilot MD, Benyahya L, Dufour A (2010) Spatial variability of atmospheric fluxes of metals (Al, Fe, Cd, Zn and Pb) and phosphorus over the whole Mediterranean from a one-year monitoring experiment: biogeochemical implications. Mar Chem 120:164–178

[CR62] Harris WR, Amin SA, Küpper FC, Green DH, Carrano CJ (2007) Borate binding to siderophores: structure and stability. J Am Chem Soc 129:12263–12271 17850151 10.1021/ja073788v

[CR63] Harrison PM, Lilley TH (1989) Ferritin. In: Loehr TM (ed) Iron carriers and iron proteins. VCH Publishers, New York

[CR64] Hasan AHH, Al-Bader DA, Woodward S, Peters AF, Küpper FC (2023) Ecophysiology of Kuwaiti macroalgae with special emphasis on temperature and salinity tolerance related to the conditions at desalination plant outfalls. Bot Mar 66:373–390

[CR65] Heimbürger LE, Migon C, Losno R, Miquel JC, Thibodeau B, Stabholz M, Dufour A, Leblond N (2014) Vertical export flux of metals in the Mediterranean Sea. Deep-Sea Res Part I-Oceanogr Res Pap 87:14–23

[CR66] Herrera-León S, Cruz C, Kraslawski A, Cisternas LA (2019) Current situation and major challenges of desalination in Chile. Desalin Water Treat 171:93–104

[CR67] Herut B, Krom MD, Pan G, Mortimer R (1999) Atmospheric input of nitrogen and phosphorus to the Southeast Mediterranean: sources, fluxes, and possible impact. Limnol Oceanogr 44:1683–1692

[CR68] Herut B, Collier R, Krom MD (2002) The role of dust in supplying nitrogen and phosphorus to the Southeast Mediterranean. Limnol Oceanogr 47:870–878

[CR69] Herut B, Zohary T, Krom MD, Mantoura RFC, Pitta P, Psarra S, Rassoulzadegan F, Tanaka T, Thingstad TF (2005) Response of East Mediterranean surface water to Saharan dust: on-board microcosm experiment and field observations. Deep Sea Res Part II Top Stud Oceanogr 52:3024–3040

[CR70] Herut B (2005) The role of desert/Sahara dust event as N and P supplier to the SE Mediterranean. In: UNEP/MAP (ed.) Atmospheric inputs of nitrogen and phosphorus to the south east Mediterranean: The role of desert/Sahara dust event as N and P supplier Athens, Greece

[CR71] Hickford SJH, Küpper FC, Zhang GP, Carrano CJ, Blunt JW, Butler A (2004) Petrobactin sulfonate, a new siderophore produced by the marine bacterium *Marinobacter hydrocarbonoclasticus*. J Nat Prod 67:1897–1899 15568785 10.1021/np049823i

[CR72] Hoffmann LJ, Peeken I, Lochte K, Assmy P, Veldhuis M (2006) Different reactions of Southern Ocean phytoplankton size classes to iron fertilization. Limnol Oceanogr 51:1217–1229

[CR73] Hopkinson BM, Barbeau KA (2012) Iron transporters in marine prokaryotic genomes and metagenomes. Environ Microbiol 14:114–128 21883791 10.1111/j.1462-2920.2011.02539.x

[CR74] Hopkinson BM, Roe KL, Barbeau KA (2008) Heme uptake by *Microscilla marina* and evidence for heme uptake systems in the genomes of diverse marine bacteria. Appl Environ Microbiol 74:6263–6270 18757577 10.1128/AEM.00964-08 PMC2570271

[CR75] Hosseini H, Saadaoui I, Moheimani N, Al Saidi M, Al Jamali F, Al Jabri H, Hamadou RB (2021) Marine health of the Arabian Gulf: drivers of pollution and assessment approaches focusing on desalination activities. Mar Pollut Bull 164:112085 33549923 10.1016/j.marpolbul.2021.112085

[CR76] Ito A (2013) Global modeling study of potentially bioavailable iron input from shipboard aerosol sources to the ocean. Glob Biogeochem Cycles 27:1–10

[CR77] John EU, Okoronkwo CO, Okorefe CO, Nwokodi NC, Anyanwu OJ (2023) Analysis of physico-chemical and microbiological parameters of ship generated wastewater from vessels in Apapa Seaport Nigeria. Ecol Conserv Sci 3:555619

[CR78] Joos F, Sarmiento JL, Siegenthaler U (1991a) Estimates of the effect of Southern Ocean iron fertilization on atmospheric CO_2_ concentrations. Nature 349:772–775

[CR79] Joos F, Siegenthaler U, Sarmiento JL (1991b) Possible effects of iron fertifilization in the Southern Ocean on atmospheric CO_2_ concentration. Glob Biogeochem Cycles 5:135–150

[CR80] Kelaher BP, Coleman MA (2022) Spatial extent of desalination discharge impacts to habitat-forming species on temperate reefs. Mar Pollut Bull 175:113368 35114545 10.1016/j.marpolbul.2022.113368

[CR81] Kelly LW, Barott KL, Dinsdale E, Friedlander AM, Nosrat B, Obura D, Sala E, Sandin SA, Smith JE, Vermeij MJA, Williams GJ, Willner D, Rohwer F (2012) Black reefs: iron-induced phase shifts on coral reefs. ISME J 6:638–649 21881615 10.1038/ismej.2011.114 PMC3280131

[CR82] KSA (2021a) Executive regulation for sustainable management of the marine and coastal environment. In: Ministry of Environment, W.A.A.M., Kingdom of Saudi Arabia (ed.). Riyadh.

[CR83] KSA (2021b) Executive regulations for the protection of aqueous media from pollution. In: Ministry of Environment, W. A. A. M., Kingdom of Saudi Arabia (ed.). Riyadh

[CR84] KSA (2020) Environmental Law (Royal Decree No. M/165). In: Ministry of Environment, W.A.A.M., Kingdom of Saudi Arabia (ed.) Royal Decree No. M/165. Riyadh

[CR85] Kudo I, Noiri Y, Imai K, Nojiri Y, Nishioka J, Tsuda A (2005) Primary productivity and nitrogenous nutrient assimilation dynamics during the Subarctic Pacific Iron Experiment for Ecosystem Dynamics Study. Prog Oceanogr 64:207–221

[CR86] Küpper FC, Carrano CJ, Kuhn JU, Butler A (2006) Photoreactivity of iron(III)-aerobactin: photoproduct structure, iron(III) coordination, and iron(III) uptake by marine *Vibrio* sp. DS40M5. Inorg Chem 45:6028–6033 16842010 10.1021/ic0604967

[CR87] Lampe RH, Coale TH, Mcquaid JB, Allen AE (2024) Molecular mechanisms for iron uptake and homeostasis in marine eukaryotic phytoplankton. Annu Rev Microbiol 78:213–232 39018471 10.1146/annurev-micro-041222-023252

[CR88] Liu X, Millero FJ (1999) The solubility of iron hydroxide in sodium chloride solutions. Geochim Cosmochim Acta 63:3487–3497

[CR89] Liu J, Chakraborty S, Hosseinzadeh P, Yu Y, Tian S, Petrik I, Bhagi A, Lu Y (2014) Metalloproteins containing cytochrome, iron-sulfur, or copper redox centers. Chem Rev 114:4366–4469 24758379 10.1021/cr400479b PMC4002152

[CR90] Liu P, Zhang H, Fan Y, Xu D (2023) Microbially influenced corrosion of steel in marine environments: a review from mechanisms to prevention. Microorganisms 11:2299 37764143 10.3390/microorganisms11092299 PMC10535020

[CR148] Lykkebo Petersen K, Heck N, Reguero BG, Potts D, Hovagimian A, Paytan A (2019) Biological and physical effects of brine discharge from the carlsbad desalination plant and implications for future desalination plant Cconstructions. Water 11(2):208.

[CR91] Madhusoodhanan R, Al-Said T, Sarkar A, Fernandes L, Ahmed A, Yamamoto T, Thuslim F, Al-Dousari A, Al-Zekri W, Al-Enezi M, Al-Ghunaim A (2024) Aeolian dust and hydro-biological characteristics: decoding dust storm impacts on phytoplankton in the northern Arabian Gulf. Sci Total Environ. 10.1016/j.scitotenv.2023.168583 37981157 10.1016/j.scitotenv.2023.168583

[CR92] Maldonado MT, Strzepek RF, Sander S, Boyd PW (2005) Acquisition of iron bound to strong organic complexes, with different Fe binding groups and photochemical reactivities, by plankton communities in Fe-limited subantarctic waters – art. No. GB4S23. Global Biogeochem Cycles. 10.1029/2005GB002481

[CR93] Marchetti A, Parker MS, Moccia LP, Lin EO, Arrieta AL, Ribalet F, Murphy MEP, Maldonado MT, Armbrust EV (2009) Ferritin is used for iron storage in bloom-forming marine pennate diatoms. Nature 457:467–470 19037243 10.1038/nature07539

[CR94] Martin JH, Fitzwater SE (1988) Iron-deficiency limits phytoplankton growth in the Northeast Subarctic Pacific. Nature 331:341–343

[CR95] Martin JH, Coale KH, Johnson KS, Fitzwater SE, Gordon RM, Tanner SJ, Hunter CN, Elrod VA, Nowicki JL, Coley TL, Barber RT, Lindley S, Watson AJ, Vanscoy K, Law CS, Liddicoat MI, Ling R, Stanton T, Stockel J, Collins C, Anderson A, Bidigare R, Ondrusek M, Latasa M, Millero FJ, Lee K, Yao W, Zhang JZ, Friederich G, Sakamoto C, Chavez F, Buck K, Kolber Z, Greene R, Falkowski P, Chisholm SW, Hoge F, Swift R, Yungel J, Turner S, Nightingale P, Hatton A, Liss P, Tindale NW (1994) Testing the iron hypothesis in ecosystems of the Equatorial Pacific Ocean. Nature 371:123–129

[CR96] Martín-Barranco A, Thomine S, Vert G, Zelazny E (2021) A quick journey into the diversity of iron uptake strategies in photosynthetic organisms. Plant Signal Behav 16:1975088 34514930 10.1080/15592324.2021.1975088 PMC8525953

[CR97] Mcquaid JB, Kustka AB, Oborník M, Horák A, Mccrow JP, Karas BJ, Zheng H, Kindeberg T, Andersson AJ, Barbeau KA, Allen AE (2018) Carbonate-sensitive phytotransferrin controls high-affinity iron uptake in diatoms. Nature 555:534–537 29539640 10.1038/nature25982

[CR146] Miller EP, Böttger LH, Weerasinghe AJ, Crumbliss AL, Matzanke BF, Meyer-Klaucke W, Küpper FC, Carrano CJ (2014) Surface bound iron: a metal ion buffer in the marine brown alga *Ectocarpus siliculosus*? J Exp Bot 65:585–594 10.1093/jxb/ert406 PMC3904714 24368501

[CR98] Minas HJ, Minas M, Packard TT (1986) Productivity in upwelling areas deduced from hydrographic and chemical fields. Limnol Oceanogr 31:1182–1206

[CR99] Moore CM, Mills MM, Milne A, Langlois R, Achterberg EP, Lochte K, Geider RJ, La Roche J (2006) Iron limits primary productivity during spring bloom development in the central North Atlantic. Glob Change Biol 12:626–634

[CR100] Müller B (2023) Iron transport mechanisms and their evolution focusing on chloroplasts. J Plant Physiol 288:154059 37586271 10.1016/j.jplph.2023.154059

[CR101] Muñoz PT, Rodríguez-Rojas F, Celis-Plá PSM, López-Marras A, Blanco-Murillo F, Sola I, Lavergne C, Valenzuela F, Orrego R, Sánchez-Lizaso JL, Sáez CA (2023) Desalination effects on macroalgae (part b):Transplantation experiments at brine-impacted sites with *Dictyota* spp. from the Pacific Ocean and Mediterranean Sea. Front Mar Sci 10:1042799

[CR102] Ncec (2021a) Environmental guideline for desalination plants (EP-M-12-V01). . In: (NCEC), N. C. F. E. C. (ed.) EP-M-12-V01. Riyadh

[CR103] Ncec (2021b) Executive regulations for the protection of aqueous media from pollution. In: (NCEC), N. C. F. E. C. (ed.) EP-M-12-V01. Riyadh

[CR104] Pan K, Lim MQ, Kraft M, Mastorakos E (2021) Development of a moving point source model for shipping emission dispersion modeling in EPISODE–CityChem v1.3. Geosci Model Dev 14:4509–4534

[CR105] Peng TH, Broecker WS (1991) Dynamic limitations on the Antarctic iron fertilization strategy. Nature 349:227–229

[CR106] Quinto OP (2024) Cuántas desalinizadoras hay en México y dónde están? Meteored

[CR107] Raiswell R, Benning LG, Tranter M, Tulaczyk S (2008) Bioavailable iron in the Southern Ocean: the significance of the iceberg conveyor belt. Geochem Trans 9:1 18513396 10.1186/1467-4866-9-7 PMC2440735

[CR108] Rosely W, Voulvoulis N (2025) When water is valued, sustainability follows: reducing leakage and promoting water reuse. Water Reuse 15(4):667–683

[CR109] Rousou M, Langeneck J, Apserou C, Arvanitidis C, Charalambous S, Chrysanthou K, Constantinides G, Dimitriou PD, García Gómez SC, Hadjieftychiou SI, Katsiaras N, Kleitou P, Kletou D, Küpper FC, Louizidou P, Martins R, Moraitis ML, Papageorgiou N, Papatheodoulou M, Petrou A, Xevgenos D, Vasiliades L, Voultsiadou E, Chintiroglou CC, Castelli A (2023) Polychaetes (Annelida) of Cyprus (Eastern Mediterranean Sea): an updated and annotated checklist including new distribution records. Diversity 15:941

[CR110] Rubin M, Berman-Frank I, Shaked Y (2011) Dust- and mineral-iron utilization by the marine dinitrogen-fixer *Trichodesmium*. Nat Geosci 4:529–534

[CR111] Ruffino B, Campo G, Crutchik D, Reyes A, Zanetti M (2022) Drinking water supply in the region of Antofagasta (Chile): a challenge between past, present and future. Int J Environ Res Public Health 19:14406 36361296 10.3390/ijerph192114406 PMC9654281

[CR112] Salinas Rodriguez SG, Schippers JC, Amy GL, Kim IS, Kennedy MD (2021) Seawater reverse osmosis desalination: assessment and pre-treatment of fouling and scaling. IWA Publishing, London

[CR113] Sánchez Munguía V (2020) La desalinizadora de agua de mar en Playas de Rosarito. Un proyecto estratégico frente a la dependencia del Río Colorado y la escasez de agua en Baja California. Norteam 15:149–172

[CR114] Sandy M, Butler A (2009) Microbial iron acquisition: marine and terrestrial siderophores. Chem Rev 109:4580–4595 19772347 10.1021/cr9002787 PMC2761978

[CR115] Schiel DR, Foster MS (2015) The biology and ecology of giant kelp forests. University of California Press, Oakland

[CR116] Scholz B, Grammatiki K, Avramidi E, Lymperaki MM, Resaikos V, Papatheodoulou M, Küpper FC (2025) First record of the diatom pathogen *Diatomophthora perforans* cf. subsp. *pleurosigmae* (Oomycota) from the Mediterranean microphytobenthos. Bot Mar 68:495–498

[CR117] Semarnat (2014) Manifestación de impacto ambiental: Planta Desalinizadora de Rosarito. Expediente 02BC2014HD027. In: Naturales, S. D. M. A. Y. R. (ed.)

[CR118] Shaked Y, Twining BS, Browning TJ, Koedooder C, Kranzler CF (2025) Trace metal biogeochemistry in the ocean: From chemical principles to biological complexity. In: Anbar A, Weis D (eds) Treatise on geochemistry. Elsevier, Oxford

[CR119] Shaw EC, Gabric AJ, Mctainsh GH (2008) Impacts of aeolian dust deposition on phytoplankton dynamics in Queensland coastal waters. Mar Freshw Res 59:951–962

[CR120] Sheppard C, Al-Husiani M, Al-Jamali F, Al-Yamani F, Baldwin R, Bishop J, Benzoni F, Dutrieux E, Dulvy NK, Durvasula SRV, Jones DA, Loughland R, Medio D, Nithyanandan M, Pilling GM, Polikarpov I, Price ARG, Purkis S, Riegl B, Saburova M, Samimi-Namin K, Taylor O, Wilson S, Zainal K (2010) The Gulf: a young sea in decline. Mar Pollut Bull 60:13–38 20005533 10.1016/j.marpolbul.2009.10.017

[CR121] Shi Z, Endres S, Rutgersson A, Al-Hajjaji S, Brynolf S, Booge D, Hassellöv I-M, Kontovas C, Kumar R, Liu H, Marandino C, Matthias V, Moldanová J, Salo K, Sebe M, Yi W, Yang M, Zhang C (2023) Perspectives on shipping emissions and their impacts on the surface ocean and lower atmosphere: An environmental-social-economic dimension. Elementa: Sci Anthropocene 11:00052

[CR122] Silva N, Purcell JP (2024) Green hydrogen in Chile - an opportunity for the world. Government of Chile, Santiago de Chile

[CR123] Silva O (2025) Chilean city first in Latin America to rely entirely on desalinated seawater. United Press International (UPI) World News, 23 May 2025 at 3:20 PM

[CR124] Stepien A, Józwiak P, Gómez SCG, Avramidi E, Grammatiki K, Lymperaki M, Küpper FC, Esquete P (2024) Description of two new *Apseudopsis* species (*A. larnacensis* sp. nov and *A. salinus* sp. nov.) (Tanaidacea: Crustacea) from the Mediterranean and a biogeographic overview of the genus. PeerJ 12:e18740 39735564 10.7717/peerj.18740 PMC11674147

[CR125] Sunda WG (2006) Trace metals and harmful algal blooms. In: Caldwell LMM, Heldmaier MG, Jackson DRB, Lange WOL, Mooney SHA, Schulze JE-D, Sommer KU (eds) Anthropogenic introductions of microalgae. Springer, Netherlands

[CR126] Sutak R, Camadro J-M, Lesuisse E (2020) Iron uptake mechanisms in marine phytoplankton. Front Microbiol 11:566691 33250865 10.3389/fmicb.2020.566691 PMC7676907

[CR127] Timermans KR, Swagerman M, Nolting RF, Vanleeuwe MA, Gerringa L, Dejong JTF, Debaar HJW (1997) Availability of iron in HNLC waters of the Southern Pacific Ocean. Abstr P Am Chem Soc 213:120-GEOC

[CR128] Tolian R, Makhsoosi AH, Bushehri PK (2020) Investigation of heavy metals in the ballast water of ship tanks after and before the implementation of the ballast water convention: Bushehr Port, Persian Gulf. Mar Pollut Bull 157:111378 32658717 10.1016/j.marpolbul.2020.111378

[CR144] UNEP (1980) Protocol for the protection of the Mediterranean Sea against pollution from land-based sources. United Nations Environment Programme/Mediterranean Action Plan, Athens

[CR131] UNEP (1995) Global programme of action for the protection of the marine environment from land-based activity (GPA) 10.1016/s0025-326x(02)00169-8 12269473

[CR129] UNEP (2006) Protecting coastal and marine environments from land-based activities: global programme of action for the protection of the marine environment from land-based activities (GPA). United Nations Environment Programme, The Hague 10.1016/s0025-326x(02)00169-8 12269473

[CR132] UNEP (2017a) Regional seas conventions and protocols. In: UNEP (ed.). Nairobi

[CR130] UNEP M (2017b) Integrated Monitoring and Assessment Programme (IMAP) – Pollution and Litter Monitoring Guidelines. United Nations Environment Programme/Mediterranean Action Plan, Athens

[CR133] Van Der Merwe R, Lattemann S, Amy G (2013) A review of environmental governance and its effects on concentrate discharge from desalination plants in the Kingdom of Saudi Arabia. Desalin Water Treat 51:262–272

[CR134] Van Leeuwe MA, Scharek R, De Baar HJW, De Jong JTM, Goeyens L (1997) Iron enrichment experiments in the Southern Ocean: physiological responses of plankton communities. Deep Sea Res Part II Top Stud Oceanogr 44:189–207

[CR135] Voutchkov N (2011) Overview of seawater concentrate disposal alternatives. Desalination 273:205–219

[CR136] Vraspir JM and Butler A (2009) Chemistry of marine ligands and siderophores. In: Annual Review of Marine Science. Palo Alto: Annual Reviews 10.1146/annurev.marine.010908.163712 PMC3065440 21141029

[CR137] Ward C, Pereira RJL, Foteinis S, Renforth P (2025) Techno-economic analysis of ocean iron fertilization. Front Clim 7:1509367

[CR138] Xevgenos D, Moustakas K, Malamis D, Loizidou M (2016) An overview on desalination & sustainability: renewable energy-driven desalination and brine management. Desalin Water Treat 57:2304–2314

[CR139] Xevgenos D, Marcou M, Louca V, Avramidi E, Ioannou G, Argyrou M, Stavrou P, Mortou M, Küpper FC (2021) Aspects of environmental impacts of seawater desalination: Cyprus as a case study. Desalin Water Treat 211:15–30

[CR140] Yarimizu K, Mardones JI, Paredes-Mella J, Norambuena-Subiabre L, Carrano CJ, Maruyama F (2022) The effect of iron on Chilean *Alexandrium catenella* growth and paralytic shellfish toxin production as related to algal blooms. Biometals 35:39–51 34716889 10.1007/s10534-021-00349-2 PMC8803708

[CR141] Zeebe RE, Archer D (2005) Feasibility of ocean fertilization and its impact on future atmospheric CO_2_ levels. Geophys Res Lett 32:9

[CR142] Zhang C, Shi Z, Zhao J, Zhang Y, Yu Y, Mu Y, Yao X, Feng L, Zhang F, Chen Y, Liu X, Shi J, Gao H (2021) Impact of air emissions from shipping on marine phytoplankton growth. Sci Total Environ 769:145488 33736263 10.1016/j.scitotenv.2021.145488

[CR143] Zhang Q, Charles PD, Bendif EM, Hester SS, Mohammad S, Rickaby REM (2024) Stimulating and toxic effect of chromium on growth and photosynthesis of a marine chlorophyte. New Phytol 241:676–686 37974482 10.1111/nph.19376

